# Supplementary material for: The genome sequence of a ground beetle, Clivina fossor (Linnaeus, 1758)
Source: Wellcome Open Res. 2024 Aug 12;9:438. [Version 1] doi: 10.12688/wellcomeopenres.22898.1 (PMC11480717; doi:10.12688/wellcomeopenres.22898.1)
Supplement: Peer review history

# Reviewer report: Reviewer response for version 1

**DOI:** 10.21956/wellcomeopenres.25214.r98232

**Published:** 2024-10-15

**Authors:** Terrence Sylvester

**Affiliations:** 1 The University of Memphis, Memphis, Tennessee, USA

The authors present the genome sequence of a ground beetle, *Clivina fossor*. The authors have done an excellent job of generating the genome assembly and scaffolding it up to chromosome level. The authors follow a standard and widely accepted genome sequencing and assembly pipeline. Data are well presented and publicly available. If the authors can include the BlobToolKit GC-coverage plot before and after contaminant removal that would be ideal to show the effectiveness of the contaminant removal pipeline.

Are sufficient details of methods and materials provided to allow replication by others?

Yes

Is the rationale for creating the dataset(s) clearly described?

Yes

Are the datasets clearly presented in a useable and accessible format?

Yes

Are the protocols appropriate and is the work technically sound?

Yes

Reviewer Expertise:

Genomics

I confirm that I have read this submission and believe that I have an appropriate level of expertise to confirm that it is of an acceptable scientific standard.

# Reviewer report: Reviewer response for version 1

**DOI:** 10.21956/wellcomeopenres.25214.r98240

**Published:** 2024-10-06

**Authors:** James Galbraith

**Affiliations:** 1 The University of Edinburgh, Edinburgh, Scotland, UK

This manuscript presents a chromosomal-level genome assembly of *Clivina fossor.*

 The background gives a basic overview of the species morphology and ecology.

 The methodology is appropriate for the project described, and is explained well in a manner that would be easy to reproduce. Additionally, all the generated raw data linked to is available.

**Minor note:**

 The European Nucleotide Archive Accession linked to to this manuscript includes the genomic sequence of two endosymbioants (Wolbachia and Spiroplasma), however this manuscript indicates contamination was removed, does not mention the inclusion of their genome sequence or describe how the two endosymbionts were identified as being Wolbachia and Spiroplasma.

Are sufficient details of methods and materials provided to allow replication by others?

Yes

Is the rationale for creating the dataset(s) clearly described?

Yes

Are the datasets clearly presented in a useable and accessible format?

Yes

Are the protocols appropriate and is the work technically sound?

Yes

Reviewer Expertise:

Comparative genomics, bioinfomatics, evolutionary biology.

I confirm that I have read this submission and believe that I have an appropriate level of expertise to confirm that it is of an acceptable scientific standard.
